# Towards an optimized inhibition of liver stage development assay (ILSDA) for *Plasmodium falciparum*

**DOI:** 10.1186/1475-2875-12-394

**Published:** 2013-11-05

**Authors:** Xiaoyan Zou, Brent L House, Michael D Zyzak, Thomas L Richie, Vincent R Gerbasi

**Affiliations:** 1 US Military Malaria Vaccine Program, Naval Medical Research Center, Silver Spring, Maryland, USA

**Keywords:** ILSDA, Sporozoite, Functional assay, Antibody, Liver stage, Hepatocyte, CSP

## Abstract

**Background:**

Experimental vaccines targeting *Plasmodium falciparum* have had some success in recent years. These vaccines use attenuated parasites, recombinant sporozoite proteins, or DNA and virus combinations to induce cell-mediated immune responses and/or antibodies targeting sporozoite surface proteins. To capitalize on the success of these vaccines and understand the mechanisms by which these vaccines function, it is important to develop assays that measure correlates of protection in volunteers. The inhibition of liver stage development assay (ILSDA) tests antibodies for the ability to block sporozoite development in hepatocytes. As such the ILSDA is an excellent candidate assay to identify correlates of humoral protection, particularly against the liver stage of malaria infection. In addition, the ILSDA can be used as a tool to evaluate novel sporozoite antigens for future vaccine development. Historically the ILSDA has suffered from low sporozoite infection rates, absence of standardized reagents, and the subjectivity associated with the traditional primary outcome measures, which depend on microscopy of stained hepatocyte cultures. This study worked to significantly improve sporozoite infection rates in hepatocytes, modify key steps in the assay protocol to reduce experimental variability, and demonstrate the utility of the ILSDA in testing antibodies targeting the circumsporozoite protein.

**Methods:**

Cryopreserved primary human hepatocytes, *Plasmodium falciparum* sporozoites, and circumsporozoite antibodies were used to optimize the ILSDA.

**Results:**

Inoculation of cryopreserved primary human hepatocytes with *Plasmodium falciparum* sporozoites improved liver stage development in the ILSDA compared to HCO4 cells. In the ILSDA, circumsporozoite antibodies suppressed liver stage development in cryopreserved primary human hepatocytes in a concentration-dependent manner. Antibody-mediated suppression of parasite development in the ILSDA at a 96-hour endpoint was more robust than the 24-hour endpoint.

**Conclusions:**

ILSDA performance is improved by the use of cryopreserved primary human hepatocytes, expediting interactions between sporozoites and hepatocytes, and extending the assay endpoint.

## Background

Approximately half of the world’s population is at risk of contracting malaria and there were 660,000 deaths from malaria in 2010 [1]. Additionally, malaria parasites are becoming increasingly resistant to anti-malarial drugs [2], underscoring the need for a vaccine. Malaria infections start with a blood meal from a malaria-infected mosquito. The mosquito proboscis introduces sporozoites into the skin where the sporozoites then translocate to the bloodstream. From the bloodstream the sporozoites travel to the liver sinusoid, move through Kupffer cells, and eventually invade a hepatocyte to initiate schizogony. After 7–10 days, several thousand merozoites leave each infected hepatocyte to infect red blood cells (erythrocytes) and cause symptomatic malaria. Vaccine efforts have focused on targeting the asymptomatic sporozoite and liver (pre-erythrocytic) stages of the disease as a mechanism to prevent both symptoms and transmission [3-5].

The leading vaccine candidate, RTS,S, protects from malaria challenge by inducing antibodies targeting the major surface protein of the sporozoite (circumsporozoite protein, or CSP) [6]. CSP antibodies most likely function by blocking sporozoite entry and development in hepatocytes [7,8]. Attenuated whole sporozoite vaccines also demonstrate efficacy by protecting volunteers in human challenge models and appear to induce protection through both cell-mediated and humoral immune responses [9-11]. Despite the fact that both of these experimental vaccine approaches induce antibodies binding to the parasite, there is no highly sensitive or specific humoral correlate of protection [11]. Both CSP ELISA and sporozoite IFA titers show significant correlations, but individuals with low titers following vaccination are sometimes protected, while individuals with high titers may remain susceptible.

Functional assays are considered a promising approach to developing stronger markers of protective immunity. These assays measure the ability of antibodies to interfere with normal function, such as inhibition of sporozoite motility, inhibition of sporozoite invasion (ISI), or inhibition of liver stage development (ILSDA). However, existing functional assays have been limited by lack of sensitivity, standardization, and reproducibility, by the non-specific inhibition exhibited by negative controls and by the subjectivity associated with relying on microscopy and histological stains to determine parasite viability [11]. This gap between promise and performance underscores the need for improved functional assays that quantitatively and reproducibly predict in vivo protection.

To strengthen existing functional assays, the Liver Stage Laboratory at the Naval Medical Research Center has established an in vitro *Plasmodium falciparum* liver stage culture system in cryopreserved primary human hepatocytes (CPHH) with real-time PCR-based measurement of parasite infection rates. In this study, infection and development rates in CPHH greatly exceed those observed in hepatocyte-derived cell lines such as HCO4 cells. The culture system allows performance of multiple liver stage experiments in the same host genetic background, improving experimental consistency. Here this culture system has been adapted for the ILSDA, yielding improved sensitivity and reliability relative to the classic ISI, which relies on immortalized hepatocyte lines as the host cell. As proof of principle, results in this study show that CSP monoclonal antibodies block liver stage development in a concentration-dependent manner.

This study describes the development of a new ILSDA and identifies requirements for the consistent measurement of antibody-mediated inhibition using CSP antibodies as a test reagent. The study highlights the ILSDA as a promising candidate to identify humoral correlates of protection from malaria vaccine trials.

## Methods

### Overview

The ILSDA is similar to the previously described ISI [12]. Both assays are designed to identify an activity in sera that blocks invasion into hepatocytes and subsequent development [13]. Hollingdale et al. practiced an ISI technique, whereby antibodies were introduced to hepatocyte cultures prior to inoculation of sporozoites [12]. In this study sporozoites were incubated with antibodies prior to inoculating the hepatocytes with the sporozoite-antibody mixture. In addition, the assay endpoint was delayed in this study to allow invaded sporozoites to develop and non-invaded sporozoites to senesce. Traditionally, the ISI read-out is performed a few hours to one day after sporozoite inoculation of hepatocytes. Although a shortened assay duration is convenient for increasing throughput, it can artificially augment the parasite load due to the presence of free sporozoite stages that have not yet washed out of the hepatocyte culture or senesced, resulting in a false signal. In the ILSDA described here, antibodies are incubated with sporozoites for 20 minutes at room temperature to allow an opportunity for test antibodies to bind to sporozoite proteins. The sporozoite-antibody mixture is then inoculated into a primary human hepatocyte culture. The culture is washed after three hours and again after 24 hours post-inoculation of sporozoites. The culture remains undisturbed for 72 hours after the second wash before harvesting cells, isolating the total RNA, and performing quantitative real-time PCR (qRT-PCR) for *Pf* 18S rRNA.

### Antibodies

Navy falciparum sporozoite antibody 1 (NFS1) was developed in-house at the Naval Medical Research Center. Rabbit anti-*Plasmodium falciparum* polyclonal anti Heat Shock Protein 70 (HSP70) antibody was purchased from LifeSpan Biosciences, Inc (Seattle, WA).

### Plating the hepatocytes

Cryopreserved primary human hepatocytes were purchased from Celsis IVT, Inc. (Baltimore, MD). The cell plating medium was prepared by adding Torpedo™ Antibiotic Mix containing pen/strep, gentamycin, amikacin, and fungizone to InVitroGRO™ CP Medium (Celsis IVT, Inc.). Vials of hepatocytes were thawed and added to the cell medium. Hepatocytes were counted and 140 k-200 k viable cells were added to each well in LabTek® chamber slides. After a three-hour incubation period, the medium was replaced with fresh media and incubated overnight.

The HC04 human liver cell line was obtained from Dr. Jetsumon Sattabongkot and maintained in culture at 37°C and 5% CO_2_ in DMEM/F12 medium supplemented with 10% FBS and 1% penicillin-streptomycin solution. HC04 (50 k/well) cells were seeded onto ECL Cell Attachment Matrix (Millipore, Billerica, MA) coated 48-well plates or LabTek® slides and incubated overnight. Twenty-four hours after seeding the cells the wells of the plates and slides were confluent with cells and contained approximately 140 k-200 k cells/well.

### *Plasmodium falciparum* sporozoite preparation

*Plasmodium falciparum* (strain NF54)-infected *Anopheles stephensi* adult females were anesthetized by soaking with 70% ethanol followed by washing with DMEM medium containing anti-bacterial and anti-fungal antibiotics (BD Biosciences, 100 units/mL of penicillin, 100 units ug/mL of streptomycin, and 0.25 μg/mL of fungizone) and hepatocyte culture medium. Salivary gland dissection was performed by: 1 – Placing each mosquito on a glass slide; 2 – Using a dissecting microscope and two 27-gauge needles, separating the head from the thorax; 3 – Separating the two sets of salivary glands from the head and/or thorax and placing the salivary glands in hepatocyte culture medium in a microcentrifuge tube. The salivary glands were then disrupted by passing the medium through a 29.5-gauge needle and syringe. The sporozoites were subsequently counted using a haemocytometer and diluted in hepatocyte culture medium to a desired concentration.

### Infection of hepatocytes

Sporozoites were introduced into the wells containing CPHH or HC04 cells and incubated at 37°C for 3 hours to allow sporozoites to infect hepatocytes. As a positive control for anti-CSP antibodies, sporozoites were incubated for 20 minutes with NFS1 at various concentrations at room temperature prior inoculation. After the sporozoite inoculation, the Lab-Tek slides were centrifuged at 110 × *g* for 1 minute at room temperature to expedite sporozoite-hepatocyte interactions. After the three-hour incubation period, CPHH or HC04 cells were washed with fresh hepatocyte culture medium to remove non-invaded sporozoites. For the one-day assay (24 hours), CPHH were incubated overnight until harvested. For the ILSDA (96 hours), CPHH or HC04 cells were harvested on Day 4.

### Harvesting the hepatocytes

CPHH or HC04 cells were trypsinized, washed once at 835 × *g* in phosphate-buffered-saline for 5 minutes, and kept at -80°C until RNA extraction. To generate a standard curve for the qRT-PCR, sporozoites were serially diluted in hepatocyte culture medium, desired sporozoite numbers were added to hepatocyte mono-layers, and total host-cell/parasite material collected and stored at -80°C until RNA extraction.

### RNA extraction

The cells were removed from -80°C storage and a Qiagen RNeasy Mini Kit protocol was followed to extract the RNA from the cells. The extracted RNA was analysed with a NanoDrop spectrophotometer (Thermo Fisher Scientific) and then stored at either 4°C (for short-term storage) or -20°C (for long-term storage).

### Quantitative real-time PCR (qRT-PCR)

For each experiment RNA from each sample was diluted to the same concentration, added to PCR reaction plates, and converted to cDNA using the High Capacity cDNA Reverse Transcription Kit (Applied Biosystems, Foster City, CA). Reaction volumes of 20 μL were used in a Thermal Cycler (MJ Research Inc., Waltham, MA) set at the following amplification protocol: 25°C for 10 minutes; 37°C for 120 minutes; 85°C for 5 seconds; hold at 4°C. The resulting cDNA was used immediately or stored at -20°C.

The following probe and primers were used to detect parasite 18S ribosomal RNA (rRNA): Taqman® MGB Probe (Applied Biosystems, Sequence: 5′ – 6FAM - CAG GTC TGT GAT GTC C - MGBNFQ - 3′); Sequence Detection Primer 1 (Applied Biosystems, Sequence: 5′ - TAA CAC AAG GAA GTT TAA GGC AAC A - 3′); Sequence Detection Primer 2 (Applied Biosystems, Sequence: 5′ - CGC GTG CAG CCT AGT TTA TCT - 3′). A plate document on the 7500 Fast System was set up according to experimental design prior to each run. To prepare the qRT-PCR reaction plate, cDNA (diluted to 15–20 ng/mL), probe (250 nM) and primers (600 nM) were mixed with TaqMan® Fast Universal PCR Master Mix (Applied Biosystems) to a total volume of 20 μL per reaction in a MicroAmp Fast Optical 96-Well Reaction Plate (Applied Biosystems) covered with MicroAmp Optical Adhesive Film. The reaction plate was then placed in a 7,500 Fast Real-Time PCR System (Applied Biosystems) to quantify parasite 18S rRNA using the default thermal cycling conditions: Stage 1 – enzyme activation: 95°C for 20 seconds; Stage 2 (repeated 40 times) – melting at 95°C for 3 seconds, annealing and polymerization at 60°C for 30 seconds. Results were analysed using the Applied Biosystems standard PCR calculation system once each run was completed. Experimental samples were compared to the standard curve generated from host cell and sporozoite mixtures containing known numbers of sporozoites.

## Results

### Cryopreserved human hepatocytes support *Plasmodium falciparum* liver stage development

**Figure 1:**
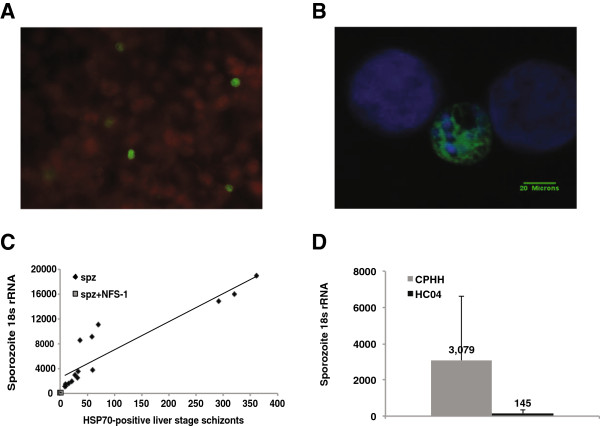
**Development of *P. falciparum *in CPHH. A**: Representative view of one 40X field typically contains several *P. falciparum (Pf)* liver schizonts. **B**: *Pf* liver stage schizont developing within a CPHH in vitro. Schizonts were stained with *mAb* anti HSP70 (purchased from LifeSpan Biosciences, Inc.) followed by fluorescein-tagged secondary antibodies (green). Nuclei were stained with DAPI (blue). Scale bar = 20 μm. **C**: Correlation between qRT-PCR *Pf* 18s rRNA copy number and counting of HSP70-positive liver stage schizonts was determined by plotting a scatter chart and linear regression (R^2^) Fifteen data points from 6 separate experiments using sporozoites alone and two data points from two separate experiments using sporozoites mixed with NFS-1 are shown. In all of the samples shown on the graph hepatocytes were analysed 96 hours after sporozoite inoculation. **D**: Comparison of sporozoite development at 96 hours after 25 k sporozoites inoculated in CPHH and HC04. Error bars represent the standard deviation of the mean.

Studying the liver stage of *P. falciparum* malaria requires a robust and reliable system of in vitro parasite cultivation. Recent efforts to cultivate *P. falciparum* liver stage sporozoites have focused on the hepatocyte-derived cell line HC04 [14]. However, HC04 cells suffer from a low *P. falciparum* sporozoite infection rate [14]. After testing multiple different cell sources, we found that cryopreserved primary human hepatocytes (CPHH) are ideal for supporting liver stage development, consistent with earlier reports using fresh primary human hepatocytes (FHH) [15]. Unlike FHH, CPHH can be obtained in large quantities, allowing for a substantial number of repeat experiments while holding host cell genotype constant. We inoculated CPHH purchased from commercial vendors with dissected *P. falciparum* sporozoites and found that CPHH with greater than 80% viability supported robust liver stage parasite development of HSP70-staining *P. falciparum* schizonts (Figure 1A and B).

### 18S rRNA qRT-PCR provides an objective, quantitative outcome measure for liver stage burden

Liver stage schizonts are often detected using immunofluorescence analysis of fixed cells (Figure 1A and B). However, these manual counting processes are subject to operator error and potential bias. To develop a more quantitative assessment of liver stage parasite biomass we measured 18S rRNAs from *P. falciparum* using qRT-PCR. Two distinct 18S rRNA genes are expressed during the *P. falciparum* lifecycle. It was shown that one 18S rRNA gene was expressed during asexual, or blood-stage malaria, while the other 18S rRNA gene was expressed during sexual, or mosquito-stage malaria. During liver stage parasite development, expression switches from the sexual to the asexual copy [16]. For the studies described here a set of primers designed to amplify both the sexual and asexual 18S rRNA was used.

To assess the extent of correlation between 18S rRNA copy number and liver stage schizonts we infected 200 K CPHH with 25 K sporozoites in six separate experiments with duplicate samples in each experiment. *Plasmodium falciparum* 18S rRNA qRT-PCR and HSP70 schizont staining were performed at 96 hours and compared in parallel, but in different samples, to avoid degradation of parasite RNA during fixation. For one of the experiments described here exceptionally high schizont yields of 300–375 HSP70-staining parasites per well were observed with 14,000-20,000 *P. falciparum* 18S rRNA copies per sample. Typically, between 10 and 70 HSP70-stained schizonts were observed per sample corresponding to 1,000-11,000 *P. falciparum* 18S rRNA copies. To suppress parasite development we treated sporozoites with 5 μg/mL of NFS1 antibodies targeting CSP prior to hepatocyte inoculation. The NFS1-treated sporozoites produced no detectable HSP70 stained schizonts and essentially no *P. falciparum* 18S rRNA signal. All of our samples were plotted with *P. falciparum* 18S rRNA levels on the Y-axis against HSP70 stained schizonts on the X-axis. A line of best fit was applied to the samples assuming a linear relationship between *P. falciparum* 18S rRNA and HSP70 stained schizonts. The coefficient of correlation between 18S copy number and HSP70 stained schizont numbers was between an R^2^ of 0.8362 and 0.95, indicating a strong correlation between ribosomal RNA levels and stained schizonts (Figure 1C). Removing the samples that had exceptionally high parasite yields (300–375 parasites per well) provided an R^2^ of 0.764. Because 18S rRNA qRT-PCR is quantitative, highly specific, and less susceptible to background effects, 18S rRNA levels using qRT-PCR were used for the remainder of the study.

### Cryopreserved primary human hepatocytes provide a significantly higher infection load compared to HC04 cells

Liver stage infection loads of 200 K CPHH or 200 K HC04 cells inoculated with 25 K dissected *P. falciparum* sporozoites were analysed by 18S rRNA qRT-PCR after 96 hours of development in vitro in 20 replicate experiments. CPHH show a 21-fold increase in parasite 18S rRNA levels compared to HC04 cells infected with the same number of sporozoites over the same incubation period (p = 3 × 10^-14^) (Figure 1D). This result suggests that CPHH display a marked improvement in parasite development compared to HC04 cells. Results presented here and those of Mazier et al. suggest that primary hepatocytes, but not hepatocyte-derived cell lines support robust parasite development [15].

### Liver stage parasite burden approaches saturation after sporozoite dose escalation

**Figure 2:**
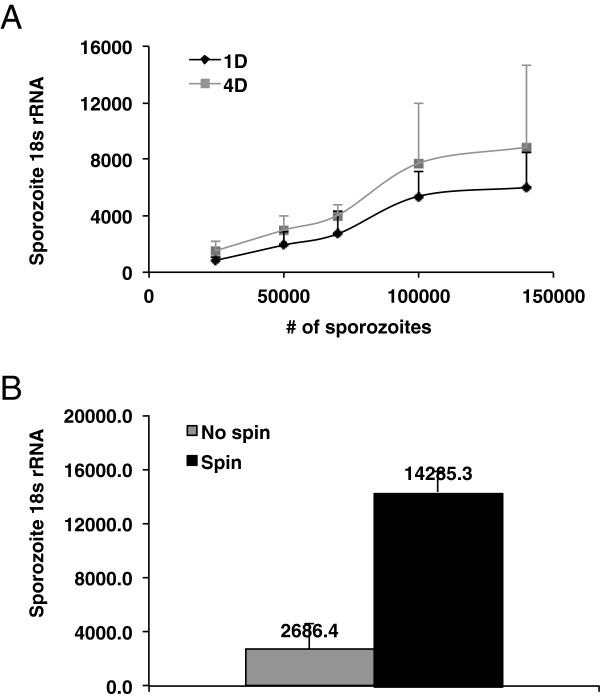
**Factors influencing *P. falciparum *liver stage development in CPHH. A**: Effect of various sporozoite numbers on infection rate of CPHH. CPHH were inoculated with 25 k, 50 k, 75 k, 100 k and 140 k *P. falciparum (Pf)* sporozoites. The liver stage parasite burden was measured by *Pf* 18s rRNA qRT-PCR on day 1 (D1) and day 4 (D4). Error bars represent the standard deviation of the mean. **B**: Effect of centrifugation on invasion rate of CPHH. CPHH were inoculated with 25 k Pf sporozoites and harvested after 96 hours. *Pf* 18s rRNA copy numbers of centrifuged samples were compared to samples without centrifugation. Error bars represent the standard deviation of the mean.

It is reasonable to speculate that incubating CPHH with an increasing load of sporozoites might result in a concomitant elevation in developed liver stage schizonts. To test this hypothesis 200 k CPHH were incubated with 25 k, 50 k, 75 k, 100 k, or 140 k *P. falciparum* sporozoites for 24 (1D) or 96 hours (4D) with media changes. From these samples parasite burden was assessed by *P. falciparum* 18S rRNA qRT-PCR. *Plasmodium falciparum* 18S rRNA levels from these samples showed a sporozoite-dependent increase in liver-stage parasite infection load (Figure 2A). These results suggest that increasing the number of sporozoites that are co-cultured with CPHH generally results in an increase in liver stage parasites. However, the liver stage parasite load appeared to reach a plateau in samples that were inoculated with 100 K-140 K sporozoites, suggesting that a saturation point was reached for liver stage development in samples that were inoculated with >100 K sporozoites. Performing a student *t*-test (equal variance 2-tails) between the samples analysed after 1 day of sporozoite inoculation of hepatocytes yielded a p = 0.0001 for 25 K sporozoites compared to 50 K sporozoites, p = 0.18 for 50 K sporozoites compared to 70 K sporozoites, p = 3.54 × 10^-6^ for 50 K sporozoites compared to 100 K sporozoites, p = 0.002 for 70 K sporozoites compared to 100 K sporozoites, and p = 0.48 for 100 K sporozoites compared to 140 K sporozoites. Performing a student *t*-test (equal variance 2-tails) between the samples analysed after 4 days of sporozoite inoculation of hepatocytes yielded a p = 0.002 for 25 K sporozoites compared to 50 K sporozoites, p = 0.09 for 50 K sporozoites compared to 70 K sporozoites, p = 0.009 for 50 K sporozoites compared to 100 K sporozoites, p = 0.13 for 70 K sporozoites compared to 100 K sporozoites, and p = 0.32 for 100 K sporozoites compared to 140 K sporozoites.

### Centrifugation enhances sporozoite invasion and liver stage development

CPHH display a complete adherence to collagen-coated slides. As a result the hepatocyte media surface is approximately 2 millimeters from the surface of the cells. Sporozoites inoculated into the hepatocyte media are buoyant and might float in the media for an extended time period before interacting with the hepatocyte target, possibly losing infectivity during the process. Previous results indicate that a brief centrifugation of the sporozoite-inoculated hepatocytes can expedite interactions between the parasite surface proteins and the hepatocytes [17]. In three replicate experiments it was observed that centrifugation after inoculation of 200 K hepatocytes with 25 K sporozoites increased liver stage parasite load by ~5.3 fold (p = 3 × 10^-4^) compared to an inoculum that was not subjected to centrifugation (Figure 2B). These results suggest that forcing sporozoite-hepatocyte interactions by centrifugation significantly elevates the liver stage parasite infection load in vitro.

### CSP antibodies suppress liver stage development in a concentration-dependent manner

**Figure 3:**
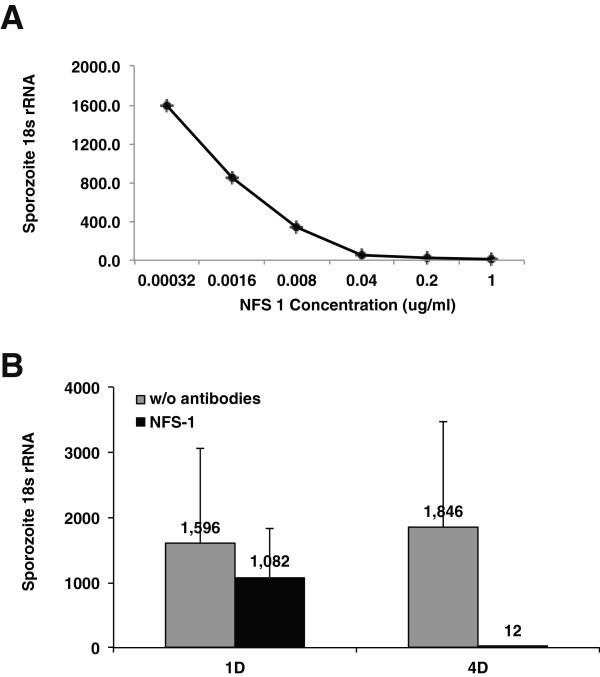
**Activity of CSP antibodies in the ILSDA. A**: CSP antibodies demonstrate concentration-dependent inhibition of liver stage development in CPHH. 25 k *P. falciparum (Pf)* sporozoites were mixed with NFS-1 at six different concentrations (0.0003, 0.0016, 0.008, 0.04, 0.2, or 1.0 μg/mL) and inoculated into CPHH. Hepatocytes were analysed after 96 hours of sporozoite inoculation. *Pf* 18s rRNA copy numbers were measured by qRT-PCR. Each data point shows the mean of a duplicate sample. **B**: Effects of NFS-1 (10 μg/mL) on the sporozoites ability to invade (1D = 1 day) and develop (4D = 4 days) in CPHH. Error bars represent the standard deviation of the mean.

Although several studies have shown that anti-CSP or anti-sporozoite antibodies suppress liver stage development in the ISI assay, few studies have demonstrated a concentration-dependent inhibition of sporozoite invasion or development [12]. To test the potential for linearity in the ILSDA, a range of CSP antibody concentrations (n = 2 at each antibody concentration) was tested. Using the anti-CSP antibody NFS1 targeting the NANP-repeat sequences of CSP, we observed a clear concentration-dependent inhibition of parasite 18S levels in the ILSDA (Figure 3A). *Plasmodium falciparum* 18S rRNA levels were not detectable when sporozoites were incubated with NFS1 antibodies at a concentration range of 0.04-1 μg/mL, suggesting that these antibodies have potent anti-sporozoite activity. Diluting the NFS1 antibody beyond a concentration of 0.04 μg/mL de-repressed liver stage development resulting in elevated *P. falciparum* 18S rRNA levels. Each five-fold dilution of NFS1 antibody resulted in an approximate 2-fold elevation in *P. falciparum* 18S rRNA levels, suggesting that the inhibitory activity of NFS1 antibodies was concentration dependent.

### Assay endpoint is critical during analysis of liver stage burden

CSP antibodies are used as positive controls in the ISI assay and ILSDA. In the ISI, investigators incubate sporozoites or hepatocytes with antibodies, inoculate the sporozoite-antibody mixture into a hepatocyte culture, and then assay for invaded sporozoites by immunostaining or qRT-PCR after 1–24 hours. The ILSDA is essentially identical to the ISI except the assay endpoint is extended from 24 to 96 hours to allow for parasite development. This study tested NFS-1 anti-CSP antibodies for liver stage growth inhibition in both the ISI (24 hour endpoint) and the ILSDA (96 hour endpoint) (Figure 3B). Using 1 μg/mL of NFS1 in the ILSDA (96-hour endpoint) resulted in a 151-fold reduction of *P. falciparum* 18S rRNA levels compared to untreated sporozoite samples (p = 5.95 × 10^-15^). In contrast, NFS1 antibodies showed only a minor reduction (<1.5-fold) in *P. falciparum* 18S rRNA levels compared to untreated control cultures in the ISI (24-hour endpoint) (p = 0.014). Collectively, these results suggest that allowing liver stage parasites to mature (96 hours) can reveal the inhibitory activity of anti-sporozoite sera. Conversely short-term incubation of sporozoites with hepatocytes in the presence of CSP antibodies (≤ 24 hours) has only a minor effect on liver stage parasite burden.

## Discussion

### Establishing a liver stage infection model in cryopreserved human primary hepatocytes

Understanding the basic science of the *P. falciparum* liver stage has been an area of research interest for decades but has been limited by a lack of a productive and robust culture system. Here a method for cultivating the *P. falciparum* liver stage using cryopreserved human hepatocytes was characterized. This system provides a higher infection rate than hepatocyte-derived cell lines. Additionally, the use of CPHH has a distinct advantage over FHH in that FHH cells represent a finite and variable resource. Because each human liver has approximately 1 × 10^11^ hepatocytes [18,19], a single CPHH donor can provide a series of repeatable experiments studying the same host/hepatocyte-parasite interaction. Collectively our results show that CPHH allow experimental repeatability with higher *P. falciparum* infection rates compared to immortalized hepatocyte cell lines.

The gold standard for detection of the *P. falciparum* liver stage involves immunostaining for exoerythrocytic forms with antibodies specific for parasite proteins. Immunostaining suffers from the subjective interpretation of what is and is not a liver stage parasite. Additionally, immunostaining liver stage parasites is less amenable to automation, which reduces the throughput of the assay. In this study we performed a correlation analysis of immunostained *P. falciparum* parasites and 18S rRNA levels. There was a strong correlation between the number of immunostained, mature liver stage schizonts and 18S rRNA levels in this study. Importantly, these results are consistent with those observed by previous studies [20,21]. Collectively, these results suggest that quantifying *P. falciparum* 18S rRNA levels can function as a substitute for counting individual liver stage parasites. Additionally, assaying parasite development by 18S rRNA qRT-PCR has the potential advantage of increased throughput and quantitation over immunostaining techniques. Finally, amplification of *P. falciparum* 18S rRNA is unambiguous which facilitates throughput and simplifies our experimental readout.

### Ways to increase productive infections in CPHH

It is natural to assume that increasing sporozoite numbers would influence the number of infected hepatocytes in vitro. Increasing the number of sporozoites that were incubated with hepatocytes resulted in an elevation in *P. falciparum* 18S rRNA levels. However, *P. falciparum* 18S rRNA levels began to saturate between 100-140 K sporozoites in wells seeded with 200 k CPHH. Importantly, this result suggests that increasing the sporozoite load will not necessarily result in a concomitant increase in the number of developing liver stage schizonts in CPHH. We hypothesize that this can be explained by several possible factors. First, it is reasonable to speculate that not all *P. falciparum* sporozoites dissected from the salivary gland have the potential to bind, invade, and develop in hepatocytes. In this scenario only sporozoites that had achieved a particular developmental profile would have the potential to establish a productive infection in a hepatocyte. Second, it is possible that the cell surface architecture of most CPHH cells is not ideal for sporozoite binding and invasion. For example, during the processing of donor liver tissue, hepatocyte surface proteins might be lost or depleted resulting in a reduction of host cell binding receptors for sporozoites. Despite the fact that we do not observe a 1:1 infection ratio of sporozoites to CPHH, this culture system appears superior to immortalized cell lines derived from hepatocytes for cultivating the liver stage of *P. falciparum.*

Consistent with previous observations, expediting the interaction between sporozoites and hepatocytes by centrifugation enhanced parasite development [17]. This result suggests that hepatocyte entry and/or subsequent development might be dependent on the proximity of the sporozoite. In this model sporozoites might benefit from an initial “dive” into the first hepatocyte until finding a final hepatocyte to initiate development. Sporozoites inoculated into culture might float for hours before precipitating to the tissue culture well bottom to interact with a hepatocyte. During this time the sporozoites could lose invasion and/or developmental potential.

### ILSDA as a quantitative assay for antibody suppression of parasite development

The ISI has been used to demonstrate strong anti-sporozoite activity in sera from RAS-protected volunteers, sera from malaria endemic areas, and monoclonal antibodies targeting CSP. These past experiments have focused on characterizing antibodies that show an exceptional inhibition of sporozoite invasion compared to non-protected or pre-immune samples [11,12,22,23]. NFS-1 antibodies targeting the CSP NANP repeat sequence suppressed *P. falciparum* development in the ILSDA in a concentration-dependent manner. Using the ISI, Hollingdale et al. used CSP monoclonal antibodies to inhibit sporozoite invasion at concentrations as low as 2.5 μg/mL [12]. Using similar CSP-targeting antibodies in the ILSDA a complete inhibition of liver stage development at concentrations as low as 0.04 μg/mL was observed. These results suggest that the ILSDA has the potential to identify the anti-sporozoite activities of non-CSP antibodies that exhibit lower activity (compared to CSP antibodies) in functional assays.

### ILSDA versus ISI and the importance of assay endpoint

Experiments employing the ISI and ILSDA have yielded conflicting results [11]. In this study the ILSDA detected strong anti-sporozoite activity from CSP antibodies (>1000-fold inhibition) compared to control antibodies. The anti-sporozoite activity of CSP antibodies in the ILSDA and the ISI were compared in parallel. While the experimental conditions of the ILSDA suggested a >1000-fold inhibition in parasite development, the ISI showed less than a 1.5-fold reduction in parasite 18S rRNA levels between samples treated with CSP antibodies compared to controls. The ILSDA and the ISI are essentially the same experiment; in both cases sporozoites are incubated with antibodies for 20 minutes at room temperature and introduced to hepatocytes, but are incubated for different periods, either 24 hours (ISI) or 96 hours (ILSDA). Therefore, the primary difference between the two assays is the co-incubation time of antibody-bound-sporozoites and hepatocytes. The difference in observed activity between the ISI and ILSDA suggests that: 1. Non-invaded sporozoites provide a background signal during amplification of the *P. falciparum* 18S rRNA, or 2. Antibodies bound to sporozoites suppress parasite development after hepatocyte invasion. Taken together, our results suggest that the ILSDA has the capacity to distinguish hepatocyte-invaded and developing parasites from non-invaded sporozoites. Because the ILSDA is more sensitive than the ISI, it should be considered a more conclusive determination of humoral anti-parasite activity.

## Conclusions

This work describes an improved method of cultivating *P. falciparum* liver stage parasites, identifies ways to enhance hepatocyte infection, and used this novel culture system to assess functional antibody responses to parasites using the ILSDA. Future work will assess the ILSDA for sensitivity and specificity in predicting protection against controlled human malaria infection in humans immunized with candidate malaria vaccines inducing protective humoral immunity.

## Competing interests

The authors declare no competing interests.

## Authors’ contributions

RVG, CZ and TLR wrote the paper. RVG, MZ, and BH served as Principal Investigator. RVG, BH, CZ, and MZ designed experiments. CZ performed experiments. CZ, RVG, MZ, and BH analysed data. All authors read and approved the final manuscript.
